# An integrated design for segmentation and classification of diabetic foot ulcers using thermography images

**DOI:** 10.1007/s40200-025-01813-3

**Published:** 2026-01-03

**Authors:** Wan Suhaimizan Bin Wan Zaki, Yamunarani Thanikachalam, Ashok Vajravelu, Prabu Rathinam, E. Thangavelu

**Affiliations:** 1 Faculty of Electrical and Electronics Engineering, Universiti Tun Hussein Onn Malaysia, BatuPahat, Johor Malaysia; 2 Department of Biomedical Engineering, KSR College of Engineering, Tiruchengode, Namakkal, Tamilnadu India; 3 Diabetologist, Monika Diabetic centre, Erode, Tamilnadu India

**Keywords:** Diabetic foot ulcers, Capsule networks, Swin transformers, Shallow network, Extreme learning principles

## Abstract

In recent days, diabetics, a chronic disease has risen significantly which leads to more health complications. Among those complications, diabetic foot ulcer (DFU) is much serious. DFU is a wound on the foot of a person who is affected with diabetics. It sometimes leads to fatality if untreated. Diagnosing the DFU in its early stage remains challenging due to medical impediments by the diabetics. Thermography serves as a promising technique in the early prediction of the DFU and aids for an improvised treatment towards the eradication of foot amputations. But still, utilizing thermography images for clinical treatments continues to be underexplored in treating DFU due to its computational complexities and existence of ambiguities in thermal images. To overcome this challenge, this research paper proposes an Intelligent Prediction System (IPS) using the modified swin transformers for an effective segmentation and deep capsule networks for an accurate prediction of DFU. In the segmentation phase, swin transformers can be used as U-NET based architecture to segment the lesions of foot ulcers. Deep features are extracted by the capsule networks and supplied to the deep shallow network which works on the standard of extreme learning networks to achieve the early prediction of DFU. The extensive experimentation is conducted using the thermal foot ulcer images in Python3.20 and Tensorflow –Keras Libraries. To verify the efficiency of the proposed schema, evaluated performances are assessed with other research experiments. Results show that the proposed schema achieves the highest prediction accuracy (99%) with promising segmented performance (98.6%). Moreover, the proposed model excels the varied residing schema and establishes a firm foothold in the early prediction of DFUs.

## Introduction

 Diabetic foot ulcer (DFU) is one of the most severe challenges of diabetes, which sometimes potentially leads to foot amputation if left untreated [1, 2]. Key indicators of DFU include changes in skin color, variations in skin temperature, foot swelling, and leg pain accompanied by dry, cracked skin. Typically, diagnosing DFU can be expensive, and delayed detection may have serious consequences. If it is not detected early, the fatality rate would increase among the patients [3–5].In contemporary scenario, several early prediction and detection methods have been proposed [6–9]. However, these methods exhibit the moderate performance with the less prediction accuracy. In addition, several intelligent approaches have also been implemented using the thermal images to detect the DFU complications automatically [10].

Several studies show that the usage of thermal imaging system can be considered as an essential technique that utilizes the foot temperature to analyse the diabetic foot complications. The thermal images exhibit intrinsic complex properties which impacts on the early prediction performance system [11]. To solve the above mentioned problem, machine learning [12] and Deep learning [13, 14] approaches are utilized as a catalyst that can boost the performance in diagnosing DFU. Several conventional ML algorithms like Support vector machines (SVM), Artificial neural networks (ANN), K-nearest neighbourhood and Hybrid Decision trees were used to predict the diabetic foots using thermal images, yet they gave poor results. Since the machine learning technique fails to detect the thermal images, this research devised deep learning as a more potential means for an early prediction of diabetic foots.

In recent days, deep learning algorithms have shown its capability in detecting the thermal images which can be used as an early prediction system. In earlier days, deep learning algorithms [15–18] were limited to an early prediction of DFU and fail to focus on the different risks level predictions of foot ulcers. To overcome this drawback, the paper proposes the integrated design of segmentation and prediction of different risk levels of diabetic foot ulcers. In this work, new prediction framework is designed by ensemble cast of powerful deep learning algorithms ssubsequently to the comprehensive testing. Besides, the proposed scheme is constructed from the base using thermal foot images. The main contribution of this article is.

1. Proposing an intelligent framework for the early prediction of risk levels of the diabetic foots relied on thermal images.
2. Proposing the novel segmentation model based on Modified Swin Transformers for the diabetic foot segmentation relied on the thermal images.
3. Assessing the highest quality hybrid schema for the deep feature extraction and high accurate prediction system based on the capsule shallow networks.
4. Evaluating the proposed model with the thermal foot image datasets and comparing with the other, existing frameworks in terms of prediction performance.

## Literature review

An enhanced UNet model-based foot ulcer segmentation approach was presented by L. Xing et al. in 2022. The suggested approach added an SVM to the output node and a coarse localization module that makes decisions relied on prior information to the traditional network UNet as its foundation. In terms of dice score (89.02%), this framework performed better but the computational complexity remained to be unsolved [19]. Mahbod et al. (2022) introduced an ensemble method based on LinkNet and U-Net, two encoder-decoder CNN models. The performance of this framework in terms of dice score is average (92.07%). But this framework consumes more memory space, which is its biggest flaw [20].

Bouallal et al. (2022) proposed a deep learning-based segmentation approach for diabetic foot (DF) thermal images, addressing challenges such as image ambiguity and low clarity. Their model, Double Encoder-ResUnet (DE-ResUnet), integrates residual networks and U-Net architecture while fusing RGB and thermal information for enhanced accuracy. Using a dataset of 398 paired thermal and RGB images from 54 healthy subjects and 145 diabetic patients, the model was trained on 50% of the data, validated on 25%, and tested on the remaining 25%. The proposed model demonstrated superior segmentation performance, achieving an average intersection over union (IoU) of 97% and effectively delineating high-risk ulceration regions such as toes and heels. This study highlights the potential of deep learning in automating DF segmentation for early diagnosis and clinical applications [21]. Munadi et al. (2022) proposed a deep learning framework for early detection of diabetic foot ulcers (DFU) using decision fusion and thermal imaging. The study aimed to improve upon previous DFU detection models, which achieved an accuracy of 97%. The proposed framework employed ShuffleNet and MobileNetV2 as baseline classifiers, trained on plantar thermogram datasets, and integrated their outputs using a novel decision fusion method. This approach significantly enhanced classification accuracy, achieving 100% in distinguishing DFU-positive and DFU-negative cases, representing a 3.4% improvement over baseline models. The findings highlight the effectiveness of decision fusion in deep learning-based DFU detection, outperforming traditional machine learning and state-of-the-art deep learning classifiers [22]. DFU segmentation using U-Net was presented by D. Bouallal et al. in 2020. The thermal and color images provided by the FLIR ONE Pro thermal camera are combined to train U-Net. According to the results, this multimodal strategy outperforms the uses of thermal pictures. It is more accurate to use this framework. However, it is not advised for real-time situations because as data size increases, system performance steadily declined [23].

J. Amin et al. in 2020 proposed an assortment of classifiers, including KNN, DT, Ensemble, softmax, and NB, used in the classification phase to analyse the classification outcomes and opt for the efficient classifiers. Deep features are retrieved during this phase and fed to these classifiers. The high-level properties of the affected regions are shown by leveraging the gradient-weighted class activation mapping (Grad-Cam) model following classification for improved comprehension. The CNN network received the categorized photos for the purpose of locating affected areas. Although the computational complexity is increased, this framework offered improved classification accuracy [24–26].

Alzubaidi et al. (2020) introduced DFU_QUTNet, a novel deep convolutional neural network designed for the classification of diabetic foot ulcers (DFU) using a dataset of 754 foot images containing both healthy and ulcer-affected skin. Unlike traditional CNN architectures, which suffer from performance degradation when excessively deep, DFU_QUTNet increases network width while maintaining depth, facilitating better gradient propagation and feature extraction. The extracted features were used to train Support Vector Machine (SVM) and K-Nearest Neighbors (KNN) classifiers. The study also compared DFU_QUTNet with fine-tuned versions of GoogleNet, VGG16, and AlexNet, demonstrating superior performance with an F1-score of 94.5%, highlighting its effectiveness in DFU classification [27].Tulloch et al. (2020) conducted a systematic review on the application of machine learning in the prevention, diagnosis, and management of diabetic foot ulcers (DFUs), highlighting its potential to enhance patient care. The study systematically analyzed 37 out of 3769 papers following PRISMA-DTA guidelines, with inclusion criteria requiring studies to mention ML, DFUs, and report relevant accuracy metrics. The review found that various ML algorithms achieved at least 90% accuracy in DFU-related tasks such as image segmentation, classification, raw data analysis, and risk assessment. Despite demonstrating promising results in controlled settings, the study emphasized the need for further research, particularly in direct comparisons with standard care practices, health economic analyses, and large-scale data collection to improve ML applications in DFU management [28]. A deep learning-based approach for precise wound area segmentation was proposed by C. Cui et al. in 2019. This technique used convolutional neural networks (CNNs) to create probability maps by first processing input images to eliminate artefacts. The infected region is extracted from the probability maps as the last step. The issue of eliminating certain false positives is also addressed. Studies revealed that this technique can deliver excellent results in terms of segmentation accuracy and the Dice index. The increased memory usage of this framework, however, is its fundamental flaw [29].

## Proposed methodology

**Fig. 1:**
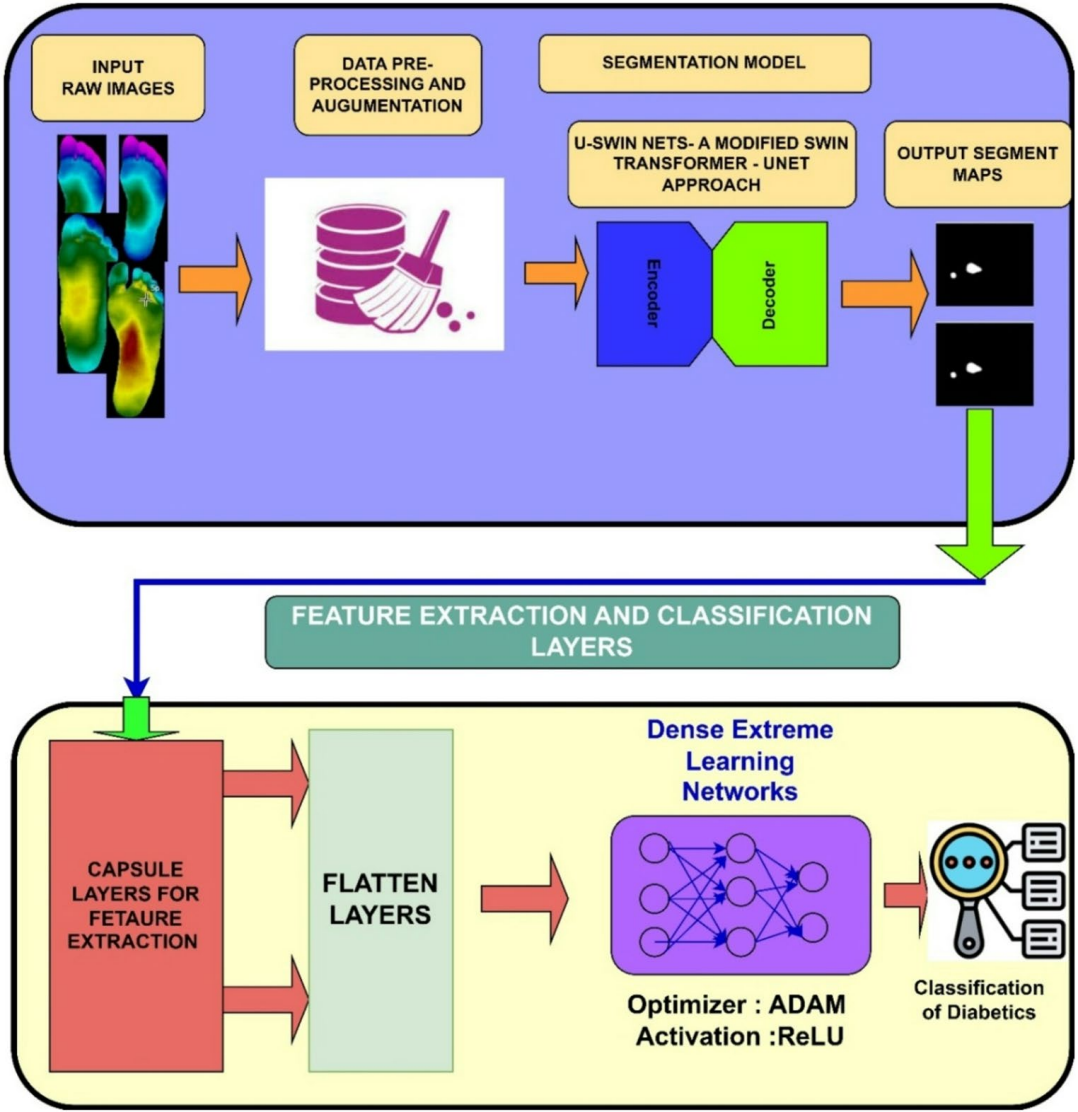
Proposed framework for segmentation, feature extraction and classification layer

In this research system, three tier architecture of hybrid deep learning framework is proposed to segment and classify the diabetic foot images. In the first stage, U-based Modified Swin transformers (U-MST) are used to segment the diabetic foot images into varied structural pathological regions. In the second stage, deep topographic features the proposed schema for integrated design of segmentation and classification is depicted in Fig. 1.

**Fig. 2:**
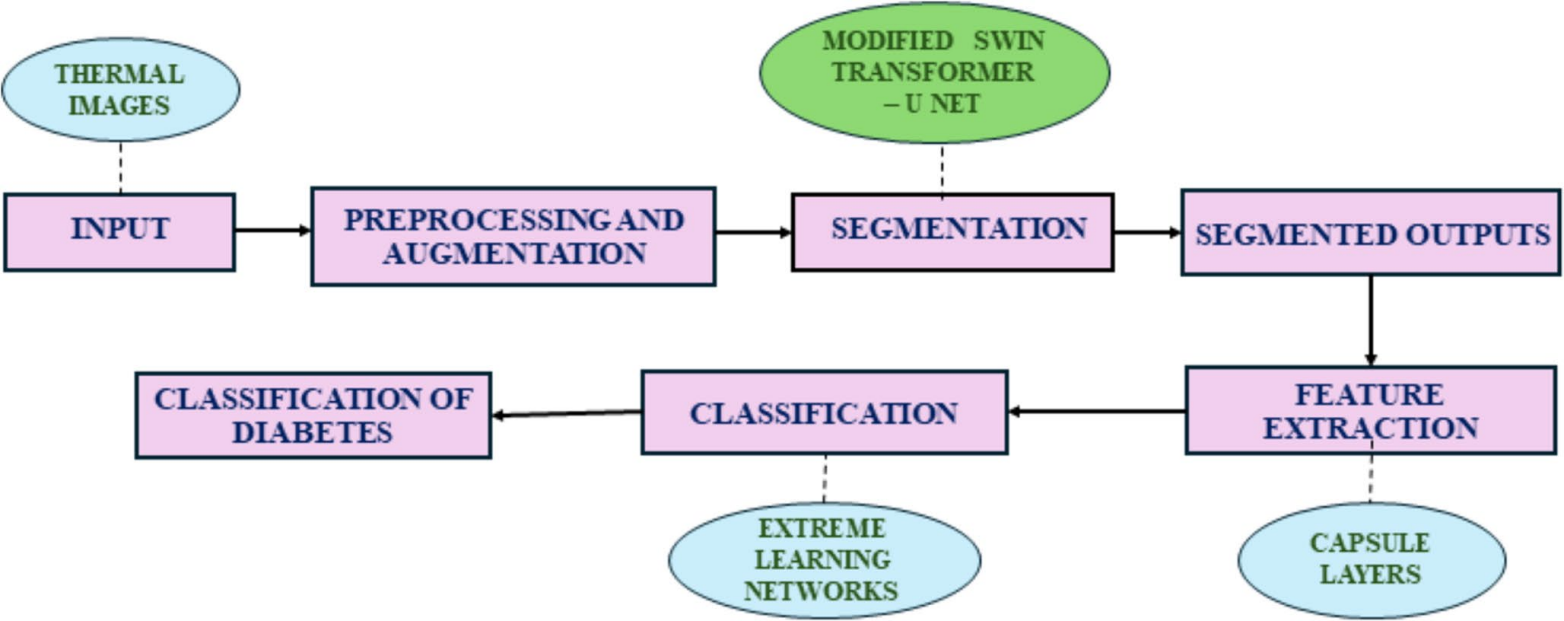
Overall framework of the proposed work

The overall framework integrates three modules — (i) Modified Swin Transformer (U-MST) for segmentation, (ii) Capsule Network for feature extraction, and (iii) Extreme Learning Machine (ELM) for final classification (Fig. 2).

The total loss function combines both segmentation and classification objectives:

L_total_ = αL_seg_ + βL_cls_​.

where L_seg_ is the Dice loss and L_cls_​ is the categorical cross-entropy. The weight coefficients α = 0.6 and β = 0.4 were empirically determined to balance optimization.

## Materials and methods

The study included patients who were clinically diagnosed with Type II diabetes mellitus for a minimum of three years, without any prior lower-limb amputation or open ulceration at the time of thermal imaging. Individuals exhibiting peripheral arterial disease, neuropathic deformities, non-diabetic foot wounds, or thermal imaging artefacts caused by skin conditions were excluded from the study. The control group consisted of healthy, non-diabetic participants with no history of vascular or neurological disorders, ensuring a clear distinction between diabetic and non-diabetic subjects for comparative analysis.

The dataset comprises thermal images (thermograms) of the foot region, sourced from [30]. It includes data collected from 122 individuals diagnosed with diabetes (DM group) and 45 non-diabetic participants (control group). Table 1 shows the demographic details of the data group.

**Table 1:** Demographic details of the diabetic and non diabetic group

Parameter	Diabetic group (n=122)	Control group (n=45)
Mean age (years)	56.2 ± 7.4	54.8 ± 6.9
Gender (M/F)	65/57	23/22
Mean BMI (kg/m^2^)	26.8 ± 3.2	25.4 ± 2.7

**Fig. 3:**
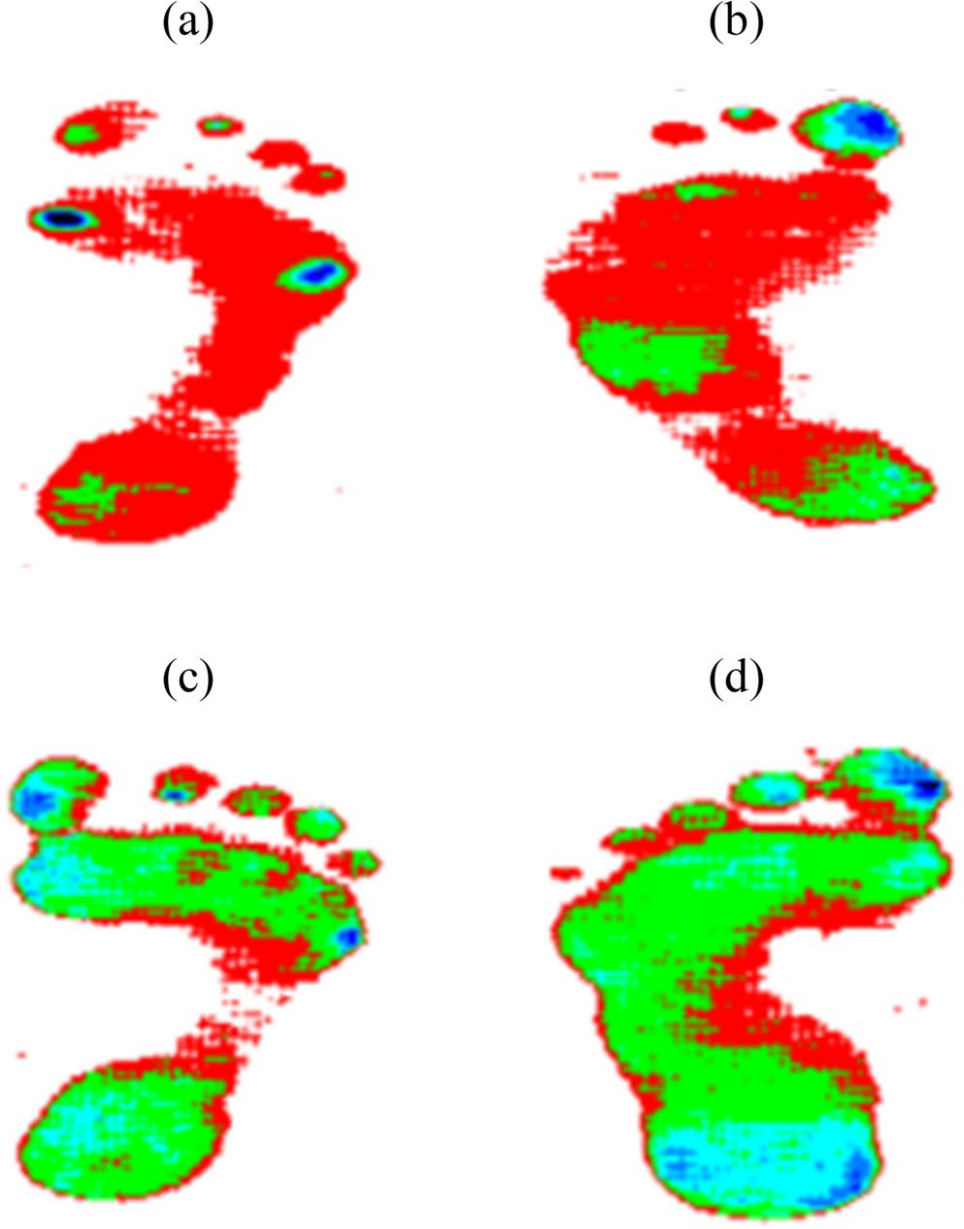
Representative thermogram images utilized for diabetic foot ulcer prediction (**a**) control group- female -right (**b**) control group- female -left (**c**) diabetic group – male -right (**d**) diabetic groups-female-left

This dataset illustrates the variations in temperature distribution within the foot area for both groups. Research indicates that elevated plantar temperature correlates with an increased risk of ulceration in diabetic individuals. The thermograms are captivated by utilizing high-resolution thermal cameras. The acquired images are systemized into folders using a distinct labelling system: ’CG’ represents the control group, while ’DM’ indicates the diabetic group. Each file is assigned a unique three-digit code, followed by a letter denoting the subject’s gender (male or female). The dataset features thermograms of both the right and left feet from subjects in the control and diabetic groups. Figure 3 illustrates the input images employed in the classification process.

### Data pre-processing

The pre-processing method is used to eliminate noise pixels and low-quality pixels that hinders the identification of foot ulcers. A pixel-intensive testing procedure has been implemented to neglect unreliable and noisy pixels from the input thermal images. Additionally, image histogram techniques are utilized to enhance image quality, as they perform effectively across various image types.

### Data augmentation

After the initial pre-processing of input images, the proposed architecture uses an image augmentation process. Neural networks often encounter overfitting issues when there is a limited labelled data available. A highly effective approach to tackle this issue is by implementing data augmentation. During this stage, every image is examined to several modifications, leading to a significant boost in the quantity of corrected training image samples. As noted in [31], affine transformations are leveraged for effective data augmentation. Methods like translation, scaling, and rotation are categorized as affine transformations. Generally, the training image samples produced from the augmentation process display some level of correlation; therefore, this step is advisable to mitigate overfitting concerns.

### Cross-validation and data diversity

**Fig. 4:**
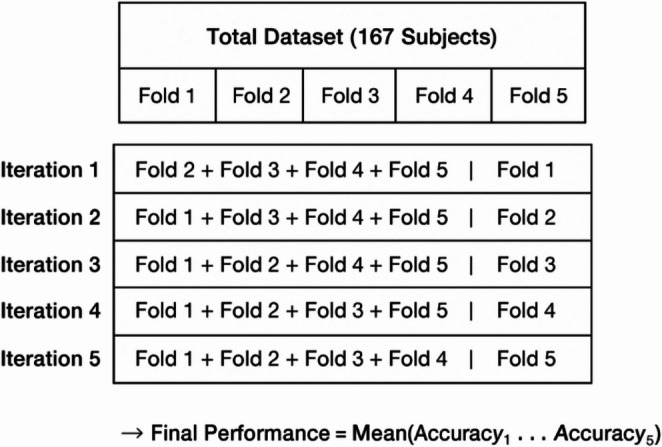
Illustration of the five-fold cross-validation strategy used in the proposed U-MST + Capsule + ELM framework

To address the limitation of the available dataset comprising 122 diabetic and 45 control subjects, a five-fold cross-validation strategy was adopted to ensure unbiased model training and reliable performance evaluation. This technique divides the entire dataset into five equal subsets (folds) of approximately equal size and distribution of diabetic and control subjects as shown in Fig. 4.

In each iteration:

1. Four folds (80%) are used for training the proposed model.
2. One fold (20%) is reserved for testing and independent evaluation.
3. The process repeats five times, ensuring that each subset serves as the test set exactly once.

After completing all five iterations, the average of the five test performances (Accuracy, Dice Score, IoU, Precision, Recall, and F1-score) is computed to represent the model’s overall capability. This approach minimizes overfitting, ensures non-overlapping subject data between training and testing, and provides a more robust estimate of model generalization, particularly important for medical datasets with limited samples.

### Segmentation model

**Fig. 5:**
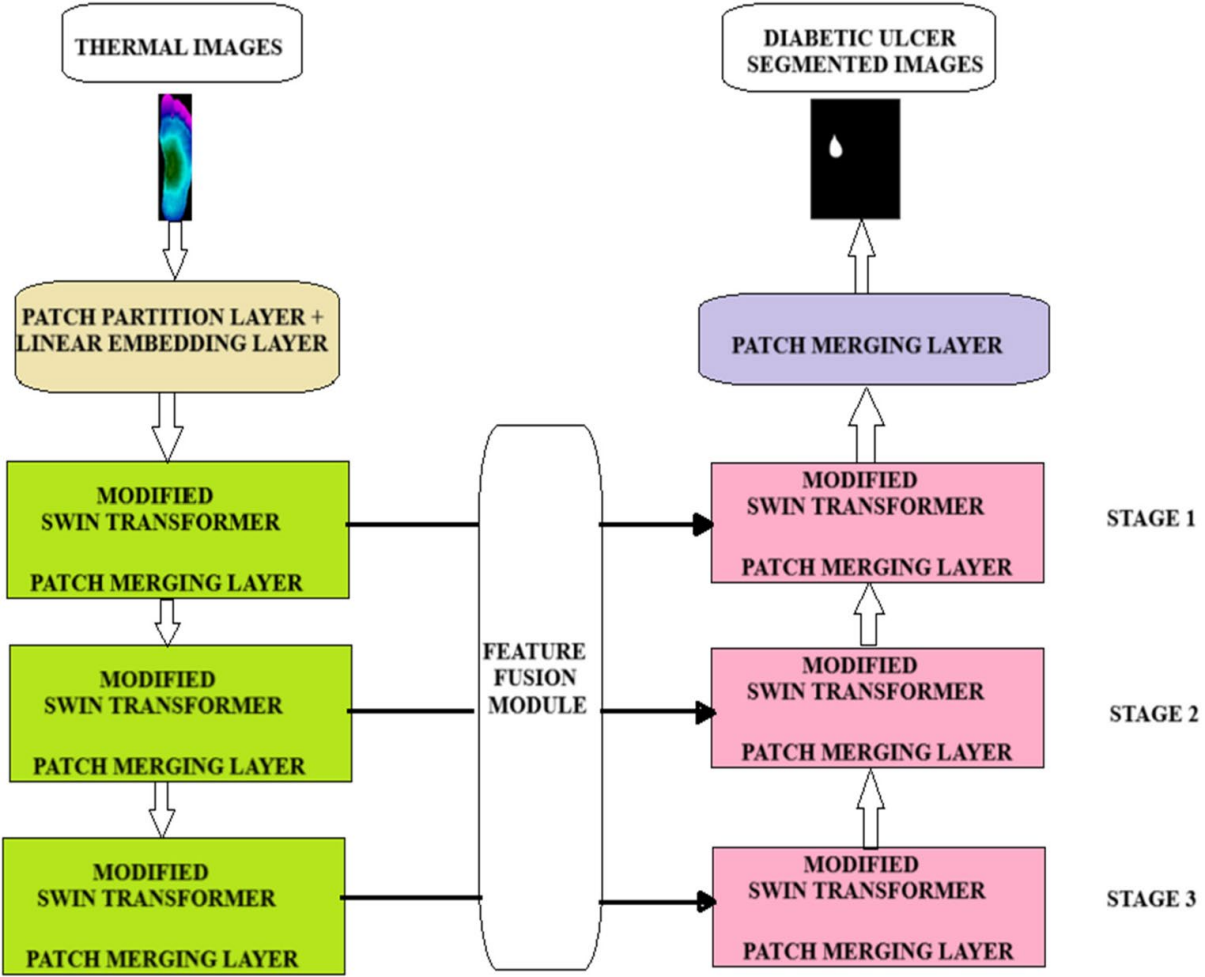
Proposed segmentation model in swin-CAP-DF NETS

The segmentation model incorporated as the backbone architecture for the segregation of diabetic thermal foot images is depicted in Fig. 5. A Modified Swin Transformer utilizing encoder-decoder architecture is introduced to achieve precise segmentation of diabetic thermal images. This architecture incorporates a feature fusion module at the end of the encoder to maximize the utilization of features across different levels.In this framework, feature maps are aggregated to enhance the transferable local attributes from various stages through multilevel feature fusion. This enables the proposed framework to localize the different ulcers, thus improving segmentation and classification accuracy. In addition, hybrid convolutional attention module (HCAM) is inserted into the modified swin transformer to support for dense building segmentation. Finally, similar architecture is incorporated as the final decoder.

### Swin transformer model

**Fig. 6:**
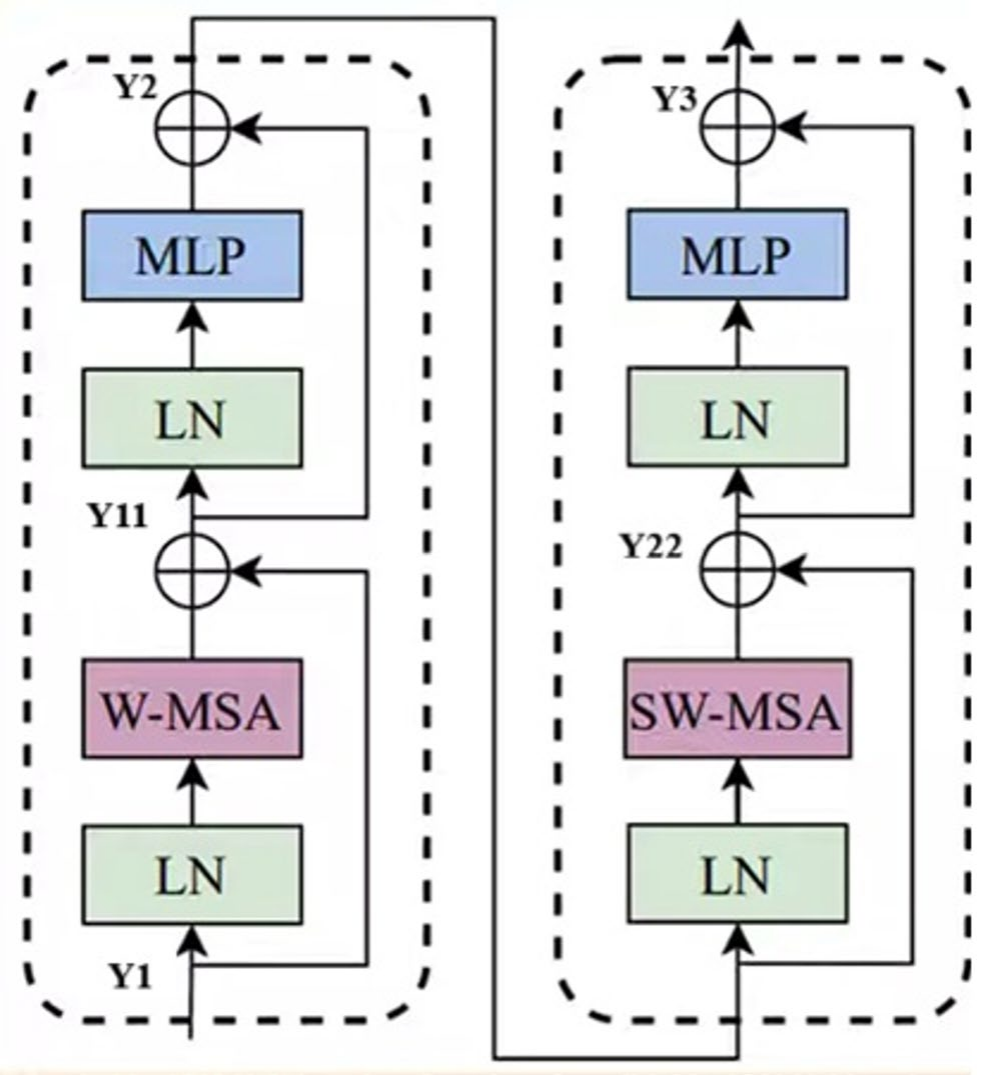
Framework for swin transformer schema

The schematic representation of the Swin Transformer module is represented in Fig. 6.

Every Swin transformer block consists of layer–normalization (LN), regular window multi-headed self attention (W-MHSA) module and multi-layer perceptron (MLP). These modules are used alternatively to form the complete swin transformer blocks. The formula for the Swin transformer block can be outlined as,

1 $$y2=W-MHDA\left(LN\left(y1\right)\right)+\left(y1-y2\right)$$

2 $$y1=MLP\left(y1\right)+\left(y1\right)$$

3 $$y1=W-MHDA\left(LN\left(y2\right)\right)+\left(y1+y2\right)$$

4 $$y2=MLP\left(y2\right)+\left(y2\right)$$

### Modified swin transformer model

**Fig. 7:**
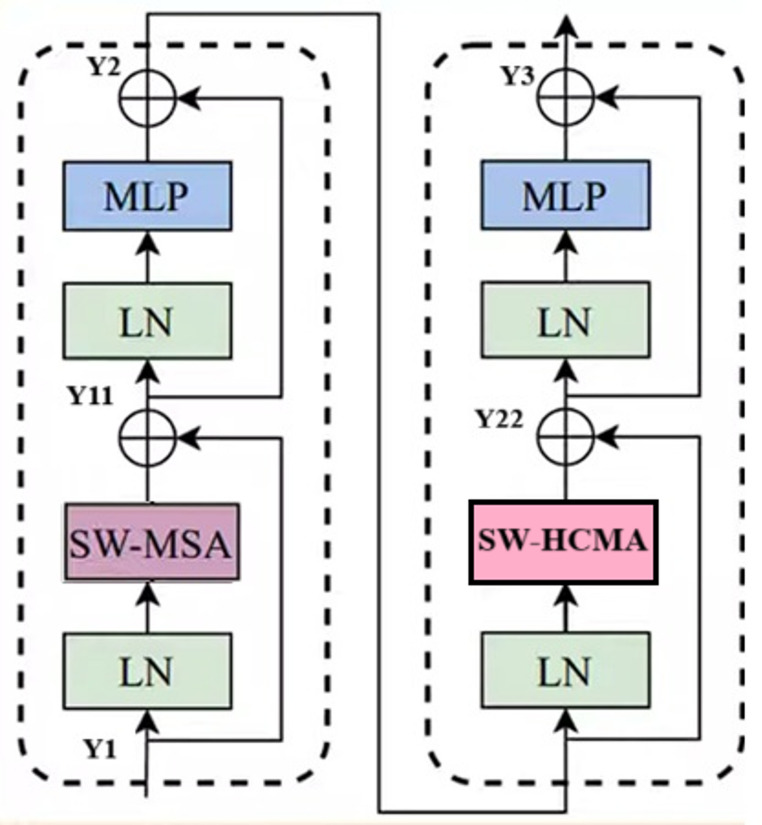
Framework of modified swin transformer schema

Figure 7 illustrates the modified swin transformer (MST) model utilized as the backbone for the proposed structure. Each MST contains a batch normalization (BN) layer, SW-MHSA, a shift window relied hybrid multi-head convolutional attention (SW-HCMA) module, a residual connection, BN layer and a MLP with residual connection. The SW-HCMA is introduced in the proposed block, which can significantly distinguish the feature maps of the thermal foot images. The SW-HCMA is constructed by combining the channel attention and spatial attention which helps to achieve the adaptive attention that focus on an accurate segmentation of the images. The proposed block integrates the Hybrid Channel-Memory Attention (HCMA) with shift window operations, mitigating adverse impacts on modelling capability, which facilitates improved accuracy. To achieve optimal performance on thermal images, the Shifted Window Multi-Head Self-Attention (SW-MHSA) and SW-HCMA are used alternately within the proposed Swin Transformer block. The mathematical formulation of the proposed Swin Transformer (SWT) can be represented as follows:

5 $$y2=W-MHSA\left(LN\left(y1\right)\right)+\left(y1-y2\right)$$

6 $$y1=MLP\left(y1\right)+\left(y1\right)$$

7 $$y3=W-HCMA\left(LN\left(y2\right)\right)+\left(y1+y2\right)$$

8 $$y2=MLP\left(y2\right)+\left(y2\right)$$

### U-SWIN core design

**Fig. 8:**
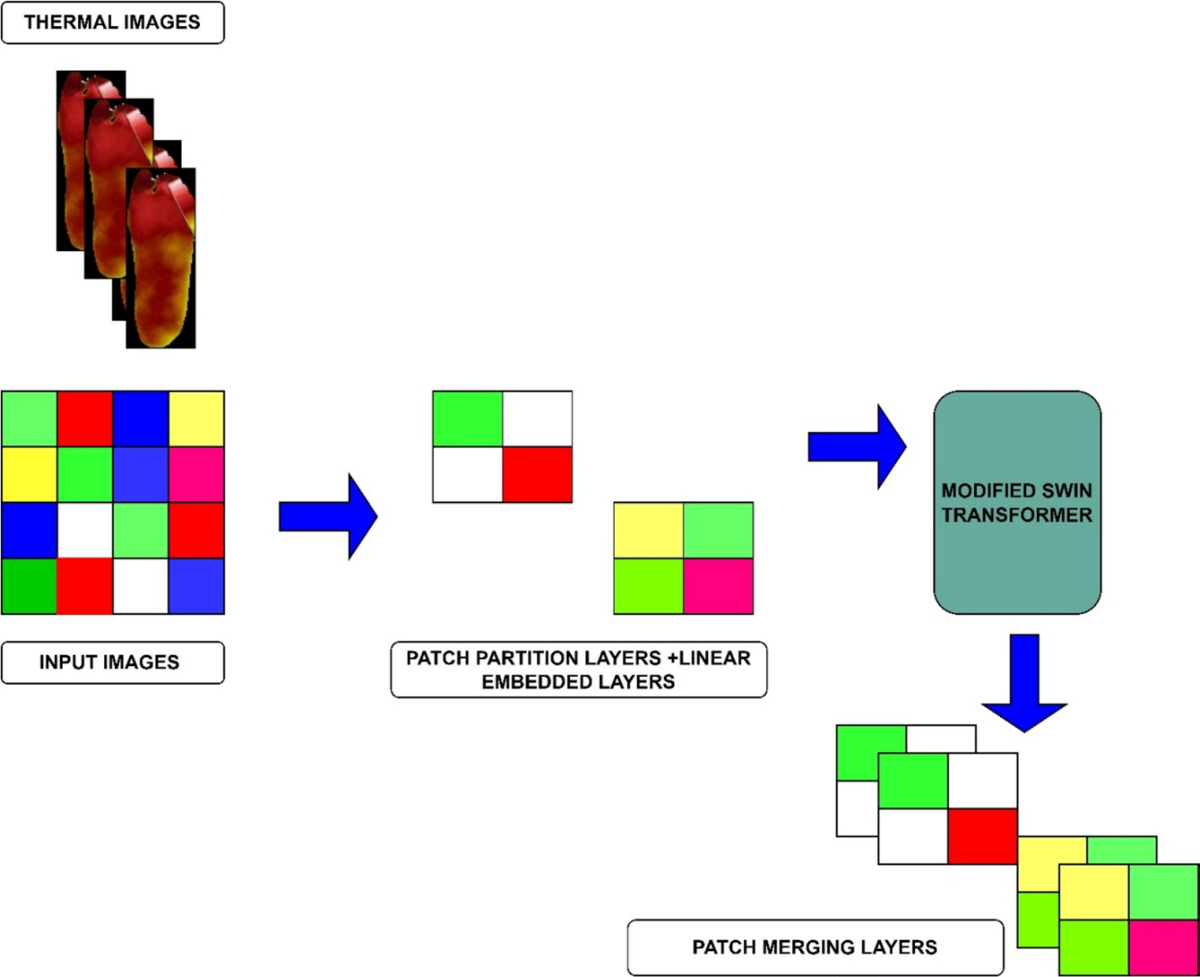
Structure of U-SWIN for handling the input thermal images in single cycle

Figure 8 shows the U-SWIN model which contains of Modified Swin transformer as a backbone with encoder, decoder and feature fusion module. The encoder-decoder design is formed as identical to the U-NET structure to handle the thermal images.

### Encoder design

In the design of the encoder, images are fed into the Patch Partition Layers (PPL) of the Modified Swin Transformer (MST), which divides them into non-overlapping patches. Every patch’s feature is constructed by concatenating the original RGB pixel values, resulting in a feature dimension corresponding to the input size. The Modified Swin Transformer Block is then utilized for hybrid attention computation, facilitating feature extraction. The Patch Merging Layers (PML) has a major impact in decreasing the number of tokens, enabling the generation of feature representations at various scales, as illustrated in Fig. 8. A combination of a patch merging layer and multiple Swin Transformer blocks is utilized to carry out feature extraction on the encoder side. Unlike the Tiny Swin Transformer model, the proposed architecture incorporates three stages, with the PPL and MST operating in Stage-1. Stage-2 and stage 3 consists of a PML module, MST blocks respectively.

### Feature fusion module

In contrast to conventional segmentation method, dataset examined for this research is thermal images with complex background with the strong refracted illuminations and different geometries. The different techniques that adopted convolutions operations in transformer provided the deeper attributes of the input images that considerably refine the segmentation quality. A feature fusion module is implemented after each phase of the Multi-Scale Transformer (MST) in the encoder. This module convolves the output feature maps and merges multi-scale features with each stage of the MST in the decoders. Despite the Swin Transformer’s hierarchical framework, there are no interactions among the feature maps at any stage. Thus, enhancing the extracted features is crucial, particularly for these unique characteristics of thermal foot images. Figure 5 showcases four feature maps from the respective stages of the Swin Transformer backbone. The flat dimension of each stage is reduced by half, while the channel dimension is increased by twofold. Each stage’s feature map is down sampled using a 2 × 2 convolutional kernel with a sliding stride of two, and then incorporated with the following stage in the channel direction.

The output from the proposed encoder is configured to match the dimensions of the feature map located on the left side of the architecture, which is denoted as O(e). Subsequently, a merging operation is executed to combine the paths. Thus, the formulation describing the interconnections among each encoder block is represented as below:

9 $$Encoder\:Stage\:Outputs\:O\left(e\right)=F\left(E\right)\varnothing F\left(dp\right)$$

For the proposed model,

10 $$O\left(e\right)=\sum\:_{i=1}^2F(E\left(i\right)\varnothing F\left(d\left(i\right)\right)$$

F(dp) is the outputs from the path merging layers.

### Decoder design

The decoder mainly consists of three stages. Contrary to the previous variants of U-net, the proposed decoder incorporates the proposed transformer block followed by the up-sampling and skip connection. The output generated by the encoder serves as the input for the decoder. In every stage of the decoder, the input features are increased in size by a factor of 2 and subsequently combined with the feature maps from the encoder at the corresponding stage, which are then processed through the MST block.

- Enables the decoder to fully leverage the characteristics from the encoder and enhance up-sampling.
- Enhances decoding efficiency.

To achieve well-defined segmented images, patch merging layers are integrated to unify features that enhance the segmentation procedure. The quantitative formulation for each stage of the decoder is presented as follows:

11 $$Decoder\:stage\:D\left(e\right)=O\left(e\right)\varnothing F\left(u\right)$$

For the Proposed model, decoder stage is given as follows,

12 $$O\left(e\right)=\sum\:_{i=1}^2F(E\left(i\right)\varnothing F\left(u\left(i\right)\right)$$

### Capsule-based feature extraction

In the subsequent phase, the images obtained from the U-SWIN module are processed through the capsule network. This Network [32] has been recently introduced to overcome the shortcomings of traditional CNN architectures. Capsules consist of clusters of neurons that represent spatial details along with the likelihood of an object’s existence. Within a capsule network, a capsule is dedicated to each entity within an image, providing:

1. Likelihood of presence in entities.
2. Parameters for instantiation of entities.

The capsule network is partitioned into three segments: a lower capsule tier, an upper capsule tier, and a classification tier. To minimize the build-up of errors, global parameter sharing is utilized, alongside an enhanced dynamic routing algorithm that updates parameters iteratively. To capture the essential spatial relationships among low-level and high-level convolutional features in the image, the product of the input vectors’ matrix and the weight matrix is computed as below:

13 $$Y\left(i.j\right)=W_{i,j}U\left(i,j\right)\ast S_j$$

14 $$S\left(j\right)={\sum\:}_jY\left(i,j\right)\ast D\left(j\right)$$

Non-linearity is ultimately introduced through the squashing function, which ensures that the vector’s direction is preserved while mapping its length to a maximum of one and a minimum of zero.

15 $$G\left(j\right)=squash(S\left(j\right)$$

To effectively classify thermal diabetic foot ulcers (DFUs), a capsule network can capture information located at various positions and establish relationships between features through the mathematical expression provided in Eq. (13). The primary capsule layers consist of convolutional layers, the specifications of which are detailed in the accompanying table. The output weights are computed using Eq. (14) and subsequently forwarded to the primary capsule region.

The squash function retains the original vector’s orientation while compressing its length to the range of (0, 1), as defined in Eq. (15). The subsequent phase integrates the dot product between similar capsules and their outputs, utilizing optimized dynamic routing. This iterative process upgrades the network’s weights to create feature maps. The dimensionality of these feature maps is then reduced via fully connected layers, transforming them into single-dimensional feature maps by utilizing flatten layers. The mathematical representation of the features is expressed in Eq. (16).

16 $$Z=F\left(G\left(j\right),P\right)$$

### Classification layers

The proposed approach leverages the principle of Extreme Learning Machines (ELM) proposed by G.B. Huang [33, 34] for rapid and precise classification of various grades. This type of neural network employs a single hidden layer, which does not require mandatory tuning. ELM utilizes a kernel function to achieve high accuracy and enhance performance. The vital benefits of ELM include minimal training error and improved approximation. With its auto-tuning of weight biases and non-zero activation functions, ELM is effectively applied in classification tasks and determining classification values. A comprehensive explanation of the ELM’s operational mechanism laid in references.

In this framework, the ‘L’ neurons in the hidden layer must utilize an activation function that is highly differentiable (such as the sigmoid function), while the output layer employs a linear activation function. The weights of the hidden layer can be allocated arbitrarily (including bias weights). Although the hidden nodes are relevant, the parameters of the hidden neurons can be randomly generated even prior to processing the training dataset. For a single-hidden layer ELM, the system output is described by Eq. (17).

17 $$f_L\left(x\right)=\:\sum\:_{i=1}^L{\beta_i\:}h_i\left(x\right)=h\left(x\right)\beta$$

Where x ◊ input features from encoder-decoder.

$\:{\upbeta\:}\:$◊ output weight vector

18 $$\beta\:=\lbrack{\beta_1,\:\beta_2,\dots\dots\dots\dots.\beta_L\rbrack}^T$$

h(x)◊ output hidden layer

19 $$h\left(x\right)=\lbrack h_1\left(x\right),h_2\left(x\right),\dots\dots\dots\dots..h_L\left(x\right)$$

20 $$H=\begin{bmatrix}h\left(x_1\right)\\\:h\left(x_2\right)\\\:\begin{array}{c}\vdots\\h\left(x_N\right)\end{array}\end{bmatrix}$$

The fundamental execution of Extreme Learning Machines (ELM) employs the simplest non-linear least squares approach, as depicted in Eq. (21).

21 $$\beta'=\:H\ast O=H^T(HH^T)^{-1}O$$

Where H∗◊ inverse of H known as Moore − Penrose generalized inverse.

22 $$\beta'=\:H^T(\frac1CHH^T)^{-1}O$$

23 $$f_L\left(x\right)=\:h\left(x\right)\beta\:=h\left(x\right)\:H^T(\frac1CHH^T)^{-1}O$$

The input feature maps, represented as h(x), form a temporal matrix, which is computed using the Moore-Penrose generalized inverse theorem. In this context, C represents a constant, while B and O signify the weights and bias parameters of the neural network. Ultimately, the likelihood of each class occurrence is determined using the softmax function, as portrayed in Eq. (24).

24 $$Y'=Softmax\left(S\right)$$

The output variable Y′ is used to forecast the diabetic foot ulcer (DFU) mechanism based on established datasets. The loss function is computed using the cross-entropy function, represented mathematically as follows:

25 $$Loss=\left(\frac1K\right)\sum\:_{i=1}^K(Y\left(i\right)\ast Log\:Y'\:+\eta\:\left|\left|\theta\right|\right|^2$$

Where K is the dimensional capsule feature length, $\eta\:\:is\:the\;regularization$ co-efficient and $\:\left|\theta\:\right|$ is the constant. The functioning process of the suggested framework is outlined in Algorithm-1.
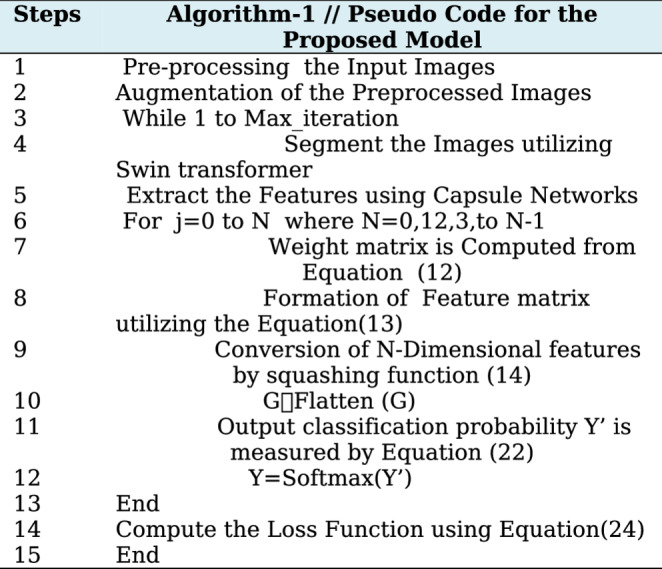


### Implementation

The network architecture introduced was formulated by utilizing Keras, with TensorFlow serving as the backend framework. Table 2 displays the hyperparameters utilized for training the schema. In training phase, the early stopping technique was utilized to halt the training process prematurely, thereby mitigating the risk of overfitting.

**Table 2:** Hyper parameters utilizing for training the proposed model

Hyperparameters used	Specifications
Initial learning rate	0.001
Epoch count	289
Batch Size	30
Optimizer	ADAM
Momentum	0.02

### Evaluation metrices

The assessment of the proposed research is conducted by utilizing two primary metrics: segmentation metrics and classification metrics. The evaluation of the segmentation model utilizes metrics to determine the disparity among the predicted outcomes and the actual ground truth. These metrics include the Dice Similarity Coefficient (DSC) and Intersection over Union (IoU). Both DSC and IoU serve as tools for measuring the overall variance between the model’s predictions and the ground truth. On the other hand, classification metrics—such as accuracy, precision, recall, specificity, and F1-score—are utilized to evaluate the proficiency of the algorithm in categorizing various levels of diabetes disorders. Table 3 provides the quantitative formulas for measuring the segmentation metrics, while Table 4 presents the equations for determining the classification metrics.

**Table 3:** Mathematical expressions for calculating the segmentation metrics

Segmentation metrics	Expression
DICE(DSC)	$\:2\left(\left|SnT\right|\right)/\left(\left|S\right|+|T\right)$
IOU	$\:\left(SnT\right)/SUT$
Segmentation precision	|SnT)/S
Segmentation recall	SnT/|S|

**Table 4:** Mathematical expression for measuring the classification metrics

Performance metrics	Mathematical expression
Accuracy	$\:\frac{TP+TN}{TP+TN+FP+FN}$
Sensitivity or recall	$\:\frac{TP}{TP+FN}\times100$
Precision	$\:\frac{TN}{TP+FP}$
F1-Score	$\:2.\frac{Precison*Recall}{Precision+Recall}$

Here, S and T denote the actual ground truth and the outcomes produced by the model. The Dice Similarity Coefficient (DSC) and Intersection over Union (IoU) values range from 0 to 1, where 0 indicates no overlap and 1 signifies a perfect match in segmentation. A greater value for these four metrics suggests an increased overlap among the model’s predictions and the actual ground truth, indicating a higher degree of similarity and improved accuracy in segmentation outcomes.

## Result and discussion

Table 5 includes recent transformer-based and hybrid deep learning models (2022–2025) relevant to medical imaging and DFU analysis. The proposed approach was assessed with the numerous advanced DL schemes in terms of performance such as respectively. Advanced deep learning approaches like TransUNet [31], Swin-UNETR [35], MedT (Gated Axial Transformer) [36], DE-ResNets [37], hybrid CNN models [38] and VIT-Caps [39] are utilized for assessment. It is vital to acknowledge that all models were trained with identical experimental parameters, utilizing the datasets organized and metrics specified in the table for analysis. The models that were trained were subsequently validated and assessed by utilizing the test data. In all instances, the datasets were partitioned, allocating 80% for training and 20% for testing purposes.

**Table 5:** Comparison with state-of-the-art transformer and hybrid

Model/Architecture	Core technique	Dataset/application	Accuracy (%)	DSC/IoU (%)	Remarks
TransUNet	CNN + Vision Transformer Hybrid	Medical lesion segmentation (retina & DFU thermography)	95.4	94.6	Combines CNN encoder with ViT decoder for improved feature attention
Swin-UNETR	Hierarchical Swin Transformer	Thermal image segmentation	96.1	95.2	Efficient hierarchical feature fusion; improved boundary delineation
MedT (Gated Axial Transformer)	Transformer with gated axial attention	Generic medical image segmentation	96.5	95.4	Improves small lesion segmentation and feature aggregation
DE-ResUNet	Double Encoder + Residual U-Net	DFU thermal image segmentation	97.0	97.0	Fusion of RGB and thermal data; improved ulcer boundary localization
Hybrid CNN-ViT (ResNet-ViT)	Residual CNN + ViT Fusion	Thermal foot ulcer recognition	97.8	96.5	Captures both global transformer context and local CNN textures
ViT-Caps	Vision Transformer + Capsule Network	Finger vein and skin ulcer classification	97.2	96.8	Integrates transformer attention with capsule routing for structural awareness

Ablation experiments were conducted to understand the contribution of each architectural component shown in Table 6. This enhanced analysis demonstrates each module’s contribution to the network’s stability and precision.

**Table 6:** Ablation experiments

Configuration	DSC (%)	IoU (%)	Accuracy (%)	Remarks
Without HCAM	94.3	92.7	95.1	Missing hybrid attention causes poor localization
Without Capsule Layer	96.8	95.4	97.0	Feature relationships under-represented
Without ELM Classifier	97.2	96.1	97.8	Slight drop in convergence speed
Full Model (Proposed)	**98.5**	**97.5**	**99.0**	**Best performance with full integration**

### Ablation analysis of the proposed algorithm (segmentation)

In this section, ablation studies were undertaken to demonstrate the impact of every element in the suggested architecture. In training phase, the variations in loss for each model were documented to assess the model’s efficacy, and the results are illustrated in Fig. 8. As illustrated, the enhanced Swin Transformer networks substantially enhanced the segmentation aspect of the model, leading to a lessened loss value throughout the training period. The ablation analysis has validated the efficacy of the proposed architecture in segregating diabetic thermal images and shows superior performance compared to other existing algorithms. Table 6 portrays the segmentation performance of the varied schemas.

### Classification results

**Fig. 9:**
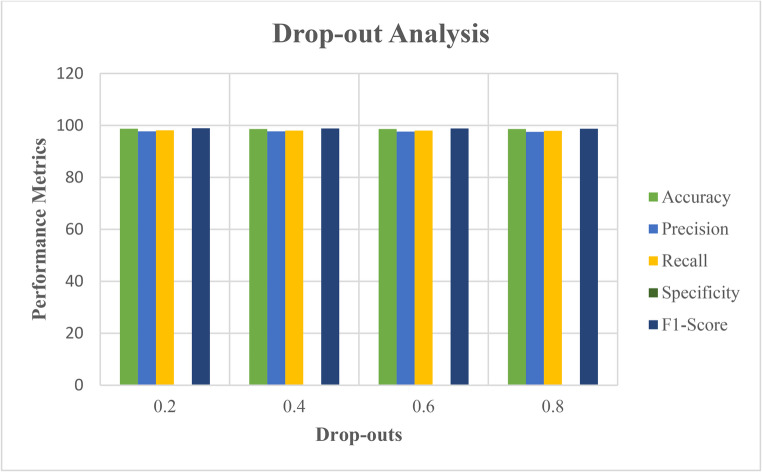
Performance validation of the recommended means with the varied drop-outs

The experimentation was carried out under two different conditions. The initial condition focused on the impact of drop-out effects on the efficacy of the proposed approach. The second scenario is calculating the classification of the advised approach and comparing with the varied residing DL algorithms in classification. Figure 9 demonstrates the effectiveness of the proposed scheme with the varied drop-out ratios. Normally the increase in the drop-outs degrades the performance of learning model. But from the Fig. 9, it is clearly shows that the effectiveness of the proposed scheme remains stable even though the drop-outs increase. The inclusion of the feed-forward layers in proposed scheme has shown the uniform effectiveness which can also overcome the over-fitting problems during training and testing (Table 7).

**Table 7:** Outcomes of segmentation for various algorithms following the ablation study utilizing test datasets

Algorithms	DSC(%)	IoU(%)	Precision(%)	Recall(%)
TransUNet	77.31%	77.80%	76.4%	73.12%
Swin-UNETR	80.24%	81.3%	80.45%	79.9%
MedT (Gated Axial Transformer)	80.6%	80.21%	78.91%	79.8%
DE-ResUNet	82.3%	82.6%	81.59%	80.5%
Hybrid CNN-ViT (ResNet-ViT)	85.09%	84.92%	83.70%	82.03%
ViT-Caps	90.74%	90.2%	90.45%	89.41%
Proposed Model	**98.5%**	**97.5%**	**97.07%**	**98.65%**

**Fig. 10:**
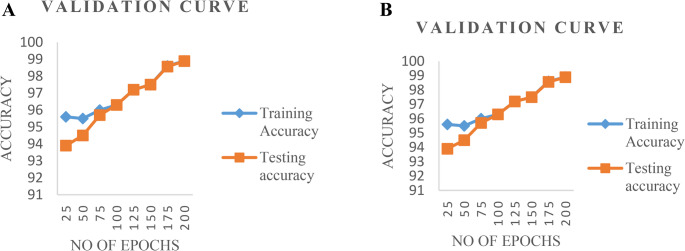
Validation performance of therecommended approach (**a**) 20% testing data (**b**) 30% training data

Moreover, the performance of the proposed schema is validated in Fig. 8. It is noticeable from Fig. 10, RMSE (root mean square error) is found very less (0.0001) between the training and testing accuracy which confirms the stability of the proposed schema in classifying the normal and sick patients from the thermal images from MRI image datasets.

Tables 8 and 9 portray the examined findings with varied schemes in classifying the thermal images. The learning model without any attention network has produced the least classification performance whereas the other hybrid models proposed from [42–45] has significantly yielded superior results compared to conventional learning approaches. The integration of transformer and feed-forward layers utilizing Extreme Learning Machines (ELM) has yielded the highest classification accuracy, reaching an impressive 0.987, significantly surpassing varied approaches by a considerable margin.

**Table 8:** Average performance metrices of the cutting edge approaches for the thermal image in detecting CG subjects

Algorithms	Performance metrics				
	Accuracy	Precision	Recall	F1-score	Computational cost (sec/image)
TransUNet	0.832	0.86	0.81	0.86	0.45
Swin-UNETR	0.83	0.70	0.82	0.733	0.52
MedT (Gated Axial Transformer)	0.88	0.812	0.88	0.825	0.49
DE-ResUNet	0.81	0.85	0.84	0.89	0.58
Hybrid CNN-ViT (ResNet-ViT)	0.82	0.80	0.89	0.89	0.51
ViT-Caps	0.90	0.85	0.82	0.845	0.43
Proposed Model	**0.958**	**0.932**	**0.98**	**0.97**	**0.37**

**Table 9:** Average performance metrics of the cutting edge approaches for the thermal image in detecting DG subjects

Algorithms	Performance metrics				
	Accuracy	Precision	Recall	F1-score	Computational cost (sec/image)
TransUNet	0.86	0.78	0.89	0.83	0.46
Swin-UNETR	0.83	0.79	0.752	0.782	0.50
MedT (Gated Axial Transformer)	0.872	0.87	0.767	0.77	0.47
DE-ResUNet	0.80	0.79	0.79	0.775	0.57
Hybrid CNN-ViT (ResNet-ViT)	0.89	0.841	0.85	0.894	0.49
ViT-Caps	0.975	0.94	0.87	0.85	0.42
Proposed Model	**0.987**	**0.97**	**0.95**	**0.952**	**0.37**

The computational cost analysis highlights that, while most transformer-based and CNN-based networks require 0.45–0.58 s per image for inference, the proposed U-MST + Capsule + ELM framework achieves superior accuracy with an average cost of only 0.37 s per image, demonstrating higher computational efficiency suitable for real-time clinical deployment.

## Conclusion and future enhancement

A Novel segmentation and classification model was proposed for the diagnosis of diabetics using thermal foot images. In this research article, diabetic detection system based on capsule and modified swin transformer was proposed. Furthermore, the article combines the benefits of Swin transformer in examining the foundational perspective comes with several benefits of capsule networks integrated with dense feed forward training networks to aid for the better segmentation and classification. Thorough tests were conducted utilizing thermography datasets, evaluating performance indicators like accuracy, precision, recall, and F1-score, DICE, PSNR, IoU and segmentation are evaluated. To prove the excellence of the proposed scheme, performance is examined with the varied residing Cutting edge DL approaches and existing hybrid architectures. Results demonstrate that proposed model showed significant improvements over the other CNN by achieving the prediction performance of 99%, 98.6% precision, 98.4%recall, 99% specificity and 99% F1-score respectively. The proposed model endowed with a novel ensemble deep learning architecture, which provided the brighter light in the direction of diagnosis system. Although the proposed system achieved a superior segmentation and prediction accuracy of 99%, future research will aim to expand the dataset by incorporating multi-center and multi-ethnic populations to ensure wider clinical applicability. The model will also be validated using external open-access datasets to enhance its generalizability and robustness. Further work will explore model compression and quantization techniques to enable deployment on portable and low-power medical devices. Additionally, interpretability will be improved through advanced explainable AI methods such as Grad-CAM + + and SHAP to provide clearer insights into decision-making processes.

## Data Availability

The datasets used for training and evaluation of the pipeline are available upon request.
